# Street Psychiatry and the Pursuit of an Ethically Attuned Framework of Care

**DOI:** 10.1192/j.eurpsy.2026.11297

**Published:** 2026-07-31

**Authors:** J. B. French, B. R. Carr

**Affiliations:** 1 University of Florida College of Medicine, Gainesville, FL, United States; 2 Department of Psychiatry, University of Florida College of Medicine, Gainesville, FL, United States

## Abstract

**Introduction:**

Street psychiatry unfolds in conditions of precarity, where clinical practice is tested by housing insecurity, social exclusion, and systemic neglect. Traditional psychiatric models—built on continuity, diagnostic stability, and institutional infrastructure—prove inadequate in such contexts. Among people experiencing unsheltered homelessness, psychiatric suffering often manifests outside established diagnostic categories and beyond the reach of structured follow-up. In these settings, psychiatric engagement requires not only clinical improvisation but also ethical recalibration.

**Objectives:**

To (1) identify recurring domains described across multidisciplinary literature on street psychiatry, and (2) propose a conceptual framework for street-based care with attention to the ethical challenges of consent, trust, diagnostic practice, and multidisciplinary integration.

**Methods:**

A narrative review was conducted across psychiatry, global health, and medical anthropology. Database searches were supplemented by reference mining of seminal works, with attention to clinical models and ethical analysis.

**Results:**

Five recurring domains were identified: evolving forms of consent, diagnostic indeterminacy, the cultivation of trust, structural barriers, and integrated care models. Emerging approaches reposition psychiatry as one component within an ecology of support. Co-responder teams, peer-led outreach, and harm-reduction psychiatry illustrate practices that emphasize relational anchoring, dignity, and adaptability.

**Conclusions:**

Street psychiatry is not simply the extension of institutional services into marginal spaces, but a reexamination of psychiatry’s conceptual core. Where continuity dissolves and certainty falters, care must be redefined less as the delivery of treatment and more as the practice of ethical attunement. Such a psychiatry grounds itself in relational fidelity and diagnostic restraint, offering lessons for the street and, by extension, for the institutional heart of the field itself.

**Disclosure of Interest:**

None Declared

